# Masticatory biomechanics of red and grey squirrels (*Sciurus vulgaris* and *Sciurus carolinensis*) modelled with multibody dynamics analysis

**DOI:** 10.1098/rsos.220587

**Published:** 2023-02-15

**Authors:** Philip G. Cox, Peter J. Watson

**Affiliations:** ^1^ Department of Cell and Developmental Biology, University College London, London, UK; ^2^ Department of Archaeology, University of York, York, UK; ^3^ Department of Engineering, University of Hull, Hull, UK

**Keywords:** multi-body dynamics analysis, skull, masticatory muscles, bite force, red squirrel, grey squirrel

## Abstract

The process of feeding in mammals is achieved by moving the mandible relative to the cranium to bring the teeth into and out of occlusion. This process is especially complex in rodents which have a highly specialized configuration of jaw adductor muscles. Here, we used the computational technique of multi-body dynamics analysis (MDA) to model feeding in the red (*Sciurus vulgaris*) and grey squirrel (*Sciurus carolinensis*) and determine the relative contribution of each jaw-closing muscle in the generation of bite forces. The MDA model simulated incisor biting at different gapes. A series of ‘virtual ablation experiments' were performed at each gape, whereby the activation of each bilateral pair of muscles was set to zero. The maximum bite force was found to increase at wider gapes. As predicted, the superficial and anterior deep masseter were the largest contributors to bite force, but the temporalis had only a small contribution. Further analysis indicated that the temporalis may play a more important role in jaw stabilization than in the generation of bite force. This study demonstrated the ability of MDA to elucidate details of red and grey squirrel feeding biomechanics providing a complement to data gathered via *in vivo* experimentation.

## 1. Introduction

The masticatory system of mammals is a highly complex arrangement of muscles running between the skull and lower jaw that act to bring the teeth into and out of occlusion in order to accomplish the process of feeding. The size and attachment sites of the individual muscles vary considerably across mammals, broadly correlating with trophic ecology (e.g. [1–3]). Many researchers have attempted to elucidate the precise function of each masticatory muscle and to determine how they all act together to produce different jaw movements, in order to better understand the biomechanics of feeding (e.g. [4–12]).

Rodents are of particular interest with regard to feeding biomechanics owing to their unique and highly specialized masticatory apparatus [3,13,14]. All rodents exhibit a highly derived dentition comprising a single pair of enlarged, ever-growing incisors and a reduced set of cheek teeth, separated by a diastema resulting from the loss of the distal incisors, the canines and most or all of the premolars [15]. The lower jaw is shorter than the skull, such that the incisors and cheek teeth cannot be in occlusion simultaneously, thus producing two distinct feeding modes i.e. incisor gnawing and molar chewing [16]. Consequently, rodents have evolved a complex configuration of jaw adductor muscles in order to produce the set of jaw movements necessary to achieve the two different feeding modes and the movement of the mandible between them. Notably, the masseter is particularly large and multi-layered in rodents and has in most species expanded its cranial attachment area on to the rostrum in front of the orbit [17]. This expansion has been achieved in three different ways in rodents, with the resulting muscle configurations known as the sciuromorphous (squirrel-like), myomorphous (mouse-like) and hystricomorphous (porcupine-like) conditions [17–19]. These masticatory muscle morphologies were historically used as a basis for rodent classification [20,21], but more recent phylogenetic analyses based on molecular data have shown repeated evolution of all three morphotypes across the rodents [22,23].

The precise function of each masticatory muscle and how they act together to produce different feeding behaviours in rodents has been the focus of numerous studies over many decades. However, given the complexity of the rodent jaw adductor musculature, it can be difficult to elucidate the contribution of each muscle to the different parts of the feeding cycle. For a number of species, estimates of how each muscle functions individually have been derived from two-dimensional vector representations of the muscle lines of action and muscle force data derived from dissection. This technique has been used to analyse a number of murid species [24–29] as well as the mountain beaver and several sciuromorphous taxa [10]. While useful, this method of course simplifies highly complex three-dimensional muscles into two-dimensional vectors and, moreover, only considers each muscle in isolation. Muscle activation patterns during gnawing and chewing can be determined *in vivo* using electromyography (EMG) but, owing to the practical and ethical constraints of implanting electrodes in the muscles of living animals, this has only been performed on a limited number of rodent species, including the house mouse (*Mus musculus* [30–32]); brown rat (*Rattus norvegicus* [33]); golden hamster (*Mesocricetus auratus* [34]); domestic guinea pig (*Cavia porcellus* [35]); springhare (*Pedetes capensis* [36]); and mountain beaver and woodchuck (*Aplodontia rufa* and *Marmota monax* [9]).

Multi-body dynamics analysis (MDA) is a computational technique which can inform how individual adductor muscles contribute to the overall process of feeding in rodents. As a computational technique, it avoids the practical and ethical issues of *in vivo* EMG measurements, and operates at a higher level of complexity than simple two-dimensional vector calculations. MDA can model jaw geometry movements in three dimensions, represents muscles as multiple strands of varying orientations in order to capture more realistic muscle paths, and can simulate multiple muscles working simultaneously or in a coordinated manner. Therefore, MDA can be used to estimate numerous biomechanical parameters such as muscle activations, joint reaction forces, bite forces and jaw movements. It must of course be acknowledged that, as with all models, an MDA simulation can only ever be as accurate as its input data. Furthermore, even though MDA models are more complex than two-dimensional vector representations, they are still a simplification compared to the highly complex reality of the vertebrate masticatory system. Although previous studies have applied MDA to investigate feeding in various mammals [37–40], there are few published studies which have investigated rodents using MDA [41], and none that have modelled squirrel feeding.

Here, we present the first MDA models of the Eurasian red squirrel (*Sciurus vulgaris*) and eastern grey squirrel (*Sciurus carolinensis*). Grey squirrels are native to North America [42] but have been introduced to Great Britain [43], Ireland [44] and northern Italy [45], where they have replaced the red squirrel over large areas. While multiple factors, including disease [46] and reproductive rates [47], are thought to underpin the success of the greys at the expense of reds, dietary competition has also been proposed to play a role [48,49]. Thus, understanding the process of feeding in red and grey squirrels is an important preliminary step in determining whether grey squirrels have an advantage over reds in this aspect of their ecology. While important work has been previously published on the anatomy of the squirrel masticatory system [3,50,51], little is currently known about the biomechanics of feeding in this group.

The aim of this study is to determine how each masticatory muscle contributes to incisor and molar biting in red and grey squirrels. Based on previously published studies of rodent masticatory biomechanics, we have three main hypotheses:

(1) The anterior deep masseter will be an important contributor to incisor bite force.

This hypothesis follows from a two-dimensional vector analysis of the rodent masticatory system [10] in which it was shown that the extension of the anterior deep master on to the rostrum seen in sciuromorphous taxa (including *Sciurus* species) increases the efficiency of incisor biting relative to the non-sciuromorphous mountain beaver. This effect occurs owing to the highly vertical orientation of the fibres of the anterior deep masseter, which align the overall masticatory muscle resultant more closely with the vector of incisor biting, thus increasing the proportion of muscle force converted into bite force (i.e. improving the mechanical advantage).

(2) The superficial masseter will have an important role in the production of bite force.

Although more horizontally oriented than the anterior deep masseter, the superficial masseter still has a vertical component to its pull and is relatively large compared to the other jaw-closing muscles. It is thus hypothesized to be a powerful elevator of the lower jaw and an important contributor to bite force. It has been demonstrated experimentally in EMG studies of the golden hamster [34] and domestic guinea pig that the superficial masseter is indeed highly activated during jaw elevation. [35].

(3) The temporalis will have a more important role in stabilization of the jaw than in the generation of bite force.

This outcome is expected based on previously published biomechanical studies of rodents. Vector analysis of mastication in a vole and field mouse indicated the temporalis to have a posterior line of action that can moderate the anterior pull of the masseter [25]. Similarly, EMG studies of the guinea pig [35] have shown the temporalis to have a braking or compensatory role, at least during molar chewing.

These hypotheses will be tested by creating MDA models of red and grey squirrel skulls to simulate incisor biting at different gapes. We will then virtually ablate each muscle, by setting their activations to zero and analyse the impact on incisor and molar bite forces. Lastly, biting on the molar row with movement enabled at the jaw joint will be simulated to assess the role of the temporalis in jaw stabilization

## 2. Material and methods

### 2.1. Specimens

The skull and mandible of an adult female red squirrel from Tomatin, Scottish Highlands, UK, were obtained from the collections of National Museums Scotland (NMS 1995.064), and were microCT scanned separately using the X-Tek Metris system at the University of Hull. Voxels were isometric with dimensions of 0.030 mm for both the skull and mandible. The head of an adult grey squirrel (*Sciurus carolinensis*) had been scanned with microCT as part of a previous project [52,53]. Three-dimensional virtual reconstructions of the cranium and mandible for both species were created using Avizo v.2020.3 (Thermo Fisher Scientific, Waltham, MA, USA). The sex of the grey squirrel specimen was not recorded, but as neither red nor grey squirrels are sexually dimorphic in size [42,54] this was unlikely to have biased the results.

Data on masticatory muscle morphology and attachment sites for the red squirrel were derived from gross dissection of a fresh-frozen red squirrel specimen (NMS GH.209.22) from the collections of National Museums Scotland, originally from Formby, Merseyside, UK. The specimen was skinned and the muscles of mastication (superficial masseter, anterior and posterior deep masseter, anterior and posterior zygomatico-mandibularis [ZM], temporalis and medial and lateral pterygoid) were dissected from each side, weighed and frozen. The muscles were subsequently digested in 30% nitric acid for 24 h to dissolve all connective tissue before being placed under glycerol [55]. Muscle fibres were gently separated with a blunt needle and photographed. Around ten fibres per muscle were measured using ImageJ, and the mean fibre length was calculated for each masticatory muscle. The physiological cross-sectional area (PCSA) of each muscle was calculated by first dividing each muscle mass by a density of 1.0564 g cm^−3^ [56] to estimate the volume, and then dividing the volume by the average fibre length. The maximum force that could be generated by each masticatory muscle was calculated by multiplying its PCSA by an intrinsic muscle stress value of 30 N cm^−^^3^ [57]. The mean of the left and right forces for each muscle was calculated. Finally, to account for the difference in size between the dissected and scanned specimens, the muscle forces were scaled using the squared ratio of their skull lengths. The scaled forces applied to the red squirrel model are given in table 1.

**Table 1.** The maximum force of the jaw-closing muscles and their relative contribution to the total adductor muscle force. Also shown, the number of strands used to represent each muscle in the MDA model.

	red squirrel		grey squirrel		
masticatory muscle	maximum force (N)	% total adductor force	maximum force (N)	% total adductor force	no. strands
superficial masseter	6.7	16.5	10.3	18.7	4
anterior deep masseter	7.1	17.5	8.8	16.0	6
posterior deep masseter	6.8	16.8	9.5	17.2	3
anterior ZM	2.5	6.2	6.9	12.5	3
posterior ZM	3.0	7.4	2.0	3.6	3
temporalis	5.7	14.1	4.2	7.3	8
medial pterygoid	5.0	12.4	10.3	18.7	5
lateral pterygoid	3.7	9.1	3.3	6.0	3

Data on the jaw-closing muscles for the grey squirrel model were taken from previously published work on the same specimen [18], as were data on the maximum force that can be generated by each muscle (table 1, taken from [53]). It should be noted that the muscle masses and fibre lengths were derived from a single formalin-fixed specimen stained with iodine potassium iodide. Both the fixation and staining reagents are known to lead to muscle shrinkage [58], which, as well as affecting muscle volume, could have impacted the curvature of the muscle fibres. Thus the muscle forces applied to the model could be artificially low, although any reduction in maximum force value is likely to be similar across all muscles.

### 2.2. MDA modelling

**Figure 1.**
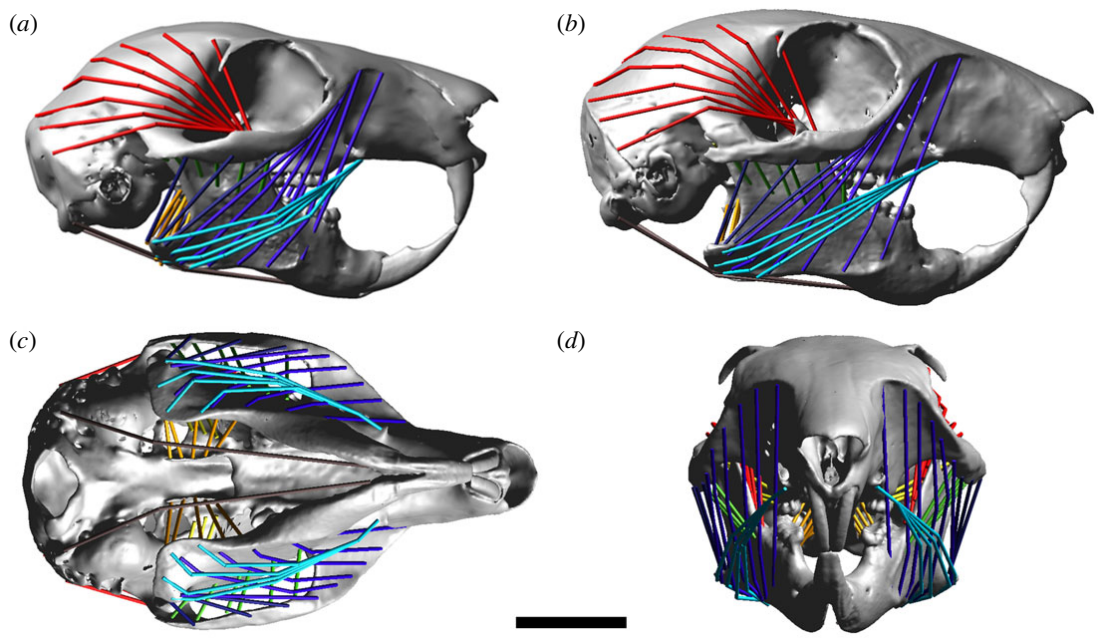
MDA model of the (*a*) red squirrel skull in right lateral view; and the grey squirrel skull in (*b*) right lateral; (*c*) ventral; and (*d*) frontal views. Muscles represented by coloured strands: sky blue, superficial masseter; royal blue, anterior deep masseter; midnight blue, posterior deep masseter; light green, anterior ZM; dark green, posterior ZM; red, temporalis; orange, medial pterygoid; yellow, lateral pterygoid; brown, digastric. Scale bar = 10 mm.

MDA models of the red and grey squirrel heads were created by importing the virtual reconstructions of the cranium and mandible to Adams View v. 2021 (MSC Software Corp., Irvine, CA, USA). The mass and inertial properties of the mandible were calculated based on volume and a standard tissue density of 1.05 g cm^−3^ [59]. The jaw-closing muscles, as listed above and in table 1, were added to the model. Each jaw adductor was modelled as a series of strands in order to capture the differing fibre directions present within a single muscle (figure 1). The masticatory system was completed by including a jaw opener (digastric muscle). Muscle wrapping was employed to enable accurate fibre excursions and to prevent muscle–bone and muscle–muscle intersections. This was particularly important for modelling the superficial masseter, anterior deep masseter, temporalis, lateral pterygoid and digastric.

Biting was simulated through modelling a food bolus positioned between the cranium and mandible on the right side. The food bolus was modelled as two rigid plates separated by a translational spring damper which connected the two plates at a coincident location. A contact with a high friction coefficient was defined between the lower plate and the mandible to ensure there was minimal displacement between the two. The translational spring damper was defined with three orthogonal forces, all of which were proportional to the distance between the two plates. The height and position of the food bolus were both adjustable in order to simulate biting at gapes.

The muscles were activated through the application of a Dynamic Geometric Optimization (DGO) method [60], which estimates the muscle forces (taking into account the instantaneous strand orientations) to make the mandible follow a specific motion (see below). Each muscle was assigned a maximum muscle force (table 1), along with a small passive tension that is naturally developed in resistance to their elongation. A maximum passive tension of 0.15 N was assigned to each muscle strand to provide resistance during jaw opening and closing [60]. This passive tension was the same in all simulations for comparative purposes. Although consideration of the length-tension curve of the masticatory muscles would provide a greater degree of accuracy in the muscle forces applied across the jaw closing cycle, this data does not currently exist for squirrels. Moreover, previous research on rats [33] concluded that changes in isometric tension over the range of muscle extension generated during feeding would be very small. We believe this holds true for the squirrel models here as the maximum muscle stretch (approximately 35% in the anterior deep masseter) is similar to that reported in the rat (30% [33]).

### 2.3. Simulation of maximum incisor biting

To test our first and second hypotheses, maximum bite forces were simulated. In order to obtain maximum bite forces that were comparable at each gape, the temporo-mandibular joint (TMJ) was modelled as a revolute joint (only permitting rotation in one degree of freedom). The DGO was set to follow a motion which opened the jaw to a distance of approximately 15.5 mm between the incisors, and then closed it again. Although no *in vivo* data on squirrel jaw motion during feeding currently exists in the published literature, studies of other rodents [34,35] suggest that, during the power stroke of incision, mandibular motion is largely constrained to the vertical axis. Thus, the use of a revolute TMJ, producing a simple hinge movement of the mandible, was felt to be a reasonable approximation of incision in the squirrel. By contrast, mastication at the molar teeth in rodents involves a much wider range of highly complex jaw movements in all three axes. Without further experimental information on squirrels, it was felt that maximal molar biting could not be realistically simulated in our MDA models and so was not included in the analyses here (but see section below on non-maximal molar biting).

To simulate a maximum bite force, the translational spring damper was set with a high stiffness in each orthogonal direction, so that the food bolus did not deform. Maximum incisor bite force was calculated using three different sizes of food bolus: 2, 7.5 and 15 mm. The largest bolus size was chosen to represent the approximate diameter of a hazelnut. The smaller sizes are an acknowledgement that squirrels do not generally bite across the widest point of a nut, but rather gnaw with multiple, smaller bites.

In order to investigate how each masticatory muscle contributes to incisor biting, maximum incisor bites were calculated in a series of simulations, whereby in each simulation the activation of each bilateral pair of muscles was set to zero. This represented a ‘virtual ablation’ of each pair of muscles, as has been undertaken previously in finite-element analysis studies (e.g. [13,61,62]). The maximum incisor bite force calculated in each simulation was then used to determine the percentage reduction in force, when compared to the maximum incisor bite force generated with all muscles active. The percentage reduction in bite force was compared across gapes to determine whether each pair of muscles performs better at narrow or wide gapes. In addition, the percentage reduction in bite force was also compared to the muscle's percentage contribution to total adductor force (table 1, as determined from muscle PCSA) to investigate if each muscle ‘overperforms’ or ‘underperforms’ relative to the theoretical maximum force it can produce.

### 2.4. Simulation of molar shearing

To test our third hypothesis, that the temporalis may play an important role in jaw stabilization during biting, non-maximal biting was simulated with contacts between the cranium and mandible at both TMJs, thus enabling the mandible to move in six degrees of freedom. The DGO was set to follow the same specified motion as in the previous simulations (i.e. create the same maximum gape), but once the jaw came into contact with the food bolus, the jaw was not constrained to any specific antero-posterior and medio-lateral path, and could move in accordance with the muscle's line of action. This simulation could not be carried out for incisor biting as the unconstrained jaw joint became too unstable. Instead, non-maximal biting was simulated at P4 and M3 (the proximal and distal extremes of the cheek tooth row, excluding the peg-like P3). The food bolus height was adjusted for P4 and M3 biting so that the gape angle at the first point of contact between the food and tooth in each case was approximately 12° in the grey, and approximately 15° in the red; these angles corresponded to the gape angles created when simulating incisor biting with a 7.5 mm food bolus. In the absence of modelling ligaments, a translational spring damper with a stiffness of 10 N mm^−1^ in all three orthogonal directions was defined at the TMJ to prevent excessive jaw movement. In order to simulate chewing of the food bolus, the translational spring damper between the rigid food plates was set to the same stiffness so that it could be compressed and sheared. It should be emphasized that the jaw movements generated in this analysis were not expected to replicate *in vivo* molar chewing in squirrels, which, as noted above, are unknown. Rather, the analysis was set up specifically to understand whether the temporalis muscle could have a role minimizing movement of the mandible.

### 2.5. Validation

Ideally, to validate a biomechanical model, its outputs should be compared with data derived from *in vivo* experiments. However, as mentioned above, no *in vivo* data has been published on squirrel feeding. Instead, the maximum incisor bite force calculated by the MDA model was compared to incisor bite force predicted from the body mass of the squirrel specimens using a published regression equation for rodents [63]. While not as good as having bite force data from *S. carolinensis* or *S. vulgaris*, the data set used to construct the regression equation does at least include the congeneric *S. niger*. The *in vivo* bite forces gathered to produce the regression equation were measured at a gape of 4.5 mm [64]. As the mass of the specimens were not directly available, this was, in turn, predicted from their skull lengths (42.8 mm and 48.2 mm for the red and grey squirrels, respectively) using a further regression equation derived from a rodent sample [65].

## 3. Results

### 3.1. Bite forces and MDA model validation

The maximum incisor bite force of the grey squirrel increased from 30.0 N at 2 mm gape, to 38.8 N at 15 mm gape. In comparison, the incisor bite forces predicted by the red squirrel MDA model were around 10 N lower: 20.1 N at 2 mm, rising to 27.0 N at 15 mm gape (table 2). The mechanical advantages (MA, calculated by dividing bite force by total adductor muscle force) for the two species were much closer in magnitude, but with the grey squirrel generally having the slightly more efficient bite. Grey squirrel MAs were 0.27–0.35 for incisor biting, whereas red squirrel MAs were 0.25–0.33.

**Table 2.** Maximum bite forces (N) and mechanical advantages (MA) at different gapes for the red and grey squirrel MDA models.

	red squirrel		grey squirrel	
gape	bite force (N)	MA	bite force (N)	MA
2 mm	20.11	0.25	30.01	0.27
7.5 mm	24.67	0.30	33.29	0.30
15 mm	26.95	0.33	38.77	0.35

The regression equations estimated body masses of 164 g and 263 g for the red and grey squirrels, respectively, leading to incisor bites of 23.4 N and 28.6 N (table 3). These values are within 5 N of the value predicted by the grey squirrel MDA model, and even closer for the red squirrel model.

**Table 3.** Skull lengths, estimated body masses [61] and estimated incisor bite forces [60] for the red and grey squirrels.

	red squirrel	grey squirrel
skull length (mm)	42.8	48.2
estimated body mass (g)	164	263
estimated bite force (N)	23.4	28.6

### 3.2. Virtual ablation

**Figure 2.**
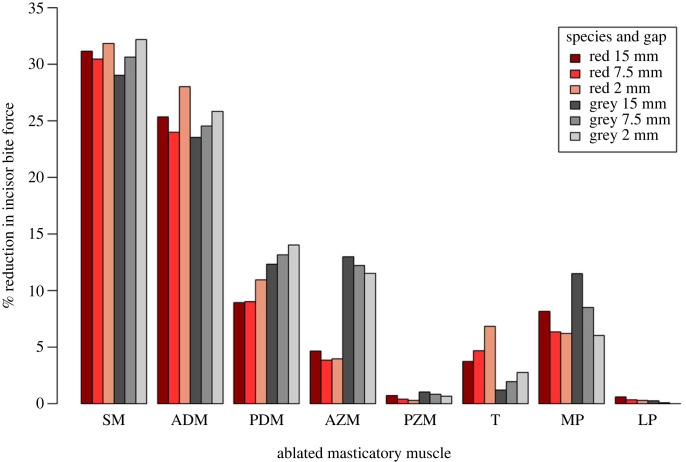
Percentage reduction in maximum incisor bite force for the red squirrel (red colours) and grey squirrel (grey colours) models with each pair of masticatory muscles set to zero activation. Darker shades represent wider gapes. ADM, anterior deep masseter; AZM, anterior zygomatico-mandibularis; LP, lateral pterygoid; MP, medial pterygoid; PDM, posterior deep masseter; PZM, posterior zygomatico-mandibularis; SM, superficial masseter; T, temporalis.

Figure 2 shows the percentage reduction in maximum incisor bite force across three gapes when the activation of each pair of masticatory muscles was set to zero. In both species, the largest reductions in bite force (over 20%) were seen with the removal of the superficial and anterior deep masseter muscles. The smallest reductions in bite force (under 5%) were found with the removal of the posterior ZM and the lateral pterygoid. The red and grey squirrels showed similar patterns in the relative importance of each masticatory muscle, except that the temporalis has a greater contribution and the anterior ZM has a reduced contribution to bite force in the red squirrel, and vice versa in the grey squirrel.

In the grey squirrel, across all gapes and bite locations analysed, it can be seen that ablation of the superficial masseter, either part of the deep masseter or the temporalis, produces a greater reduction in maximum incisor bite force at the narrower gapes (figure 2). Thus, these muscles appear to be more effective at producing bite force the closer the teeth are to occlusion. Conversely, ablation of either part of the ZM leads to a greater reduction in bite force at wider gapes. Ablation of the lateral pterygoid had a negligible effect on either bite force. These patterns are consistent between incisor and molar bites. The red squirrel shows a similar pattern for most muscles, but unlike the grey squirrel does not always show a consistent trend from wide to narrow gape. It can be seen in figure 2 that, following ablation of the superficial masseter, anterior deep masseter or anterior ZM, it is the middle gape (7.5 mm) that shows the lowest reduction in bite force.

When comparing each muscle's percentage contribution to total adductor muscle force with the reductions in maximum incisor bite force, it can be seen that the superficial masseter and the anterior deep masseter both contribute considerably more to bite force than might be expected from their PCSA. For instance, across all gapes, ablation of the superficial masseter reduces incisor bite force by 29–32% (figures 2 and 3; electronic supplementary material, tables S1–S4), despite this muscle only comprising 18.7% of the total adductor muscle force in the grey squirrel and 16.6% in the red squirrel (table 1). Similarly, the anterior deep masseter represents 16% and 17.5% of total adductor muscle force in the grey and red squirrels respectively, but its ablation reduces bite force by more than 23.5%. In comparison, other muscles (posterior deep masseter, posterior ZM, temporalis and both pterygoid muscles) all display a reduction in bite force which is less than their relative contribution to total adductor muscle force.

### 3.3. Molar shearing

Simulations of non-maximum molar biting with contacts between the cranium and mandible at the jaw joint showed that ablation of the temporalis did not significantly affect either of the P4 or M3 bite forces. However, it did very slightly increase posterior movement of the working side condyle during P4 biting, by 0.22 mm in the red squirrel, and 0.13 mm in the grey squirrel (table 4). Ablation of the temporalis also tended to move the working side condyle posteriorly during M3 biting, but, unlike in P4 biting, overall condylar movement was reduced. It was noted that ablation of the temporalis had a minimal effect on dorsoventral or medio-lateral movement of the TMJ.

**Table 4.** Bite force and movement of the working side TMJ during biting at P4 and M3 when enabling the mandible to move in six degrees of freedom. Translations are along the anterior (positive) and posterior (negative) axis.

	P4 biting		M3 biting	
red squirrel	all muscles	ablated temporalis	all muscles	ablated temporalis
bite force (N)	28.7	27.3	31.3	30.4
TMJ translation (mm)	−0.28	−0.50	0.27	−0.13
grey squirrel	al muscles	ablated temporalis	all muscles	ablated temporalis
bite force (N)	32.2	31.3	28.8	29.0
TMJ translation (mm)	−0.37	−0.50	0.26	0.05

## 4. Discussion

This study is the first to apply MDA to the skull of red and grey squirrels, thus enabling a preliminary analysis of the biomechanics of incision in these two species. In particular, the model has revealed aspects of the function of each of the jaw adductor muscles, and the impact of gape on red and grey squirrel masticatory biomechanics.

As no published *in vivo* bite forces are available for grey squirrels, the model was validated by comparing the calculated maximum incisor bite forces to those derived from a predictive regression equation relating body mass to bite force [63]. Maximum red squirrel incisor bite force values matched very closely indeed, and those of the grey squirrel were within 5 N. It is possible the MDA model slightly underestimates the true bite force as the squirrel specimens (*Sciurus niger*) in the bite force analysis tended to fall above the regression line [63], but nonetheless, we believe our model to be a close approximation of the true physiology. It should also be noted that the body mass itself was derived from skull length via a regression equation [65]. However, this second regression equation was derived from a large sample of rodents, over half of which were squirrels, and was a very strong predictor of body mass (*r*^2^ = 0.96).

It is interesting to note that the maximum bite forces calculated by the MDA model were greater with increasing gape, as has also been demonstrated in other rodents (e.g. [10,66,67]). This indicates that at least some of the squirrel masticatory muscles are more efficient at converting muscle force to bite force at wider gapes, presumably because opening the jaw increases their mechanical advantage. Indeed, on examination of the data, it appears that this is true of all the jaw adductor muscles except the temporalis. This might seem counterintuitive for muscles such as the superficial masseter which show a trend of reduced contribution to bite force as gape increases (figure 2). However, because total bite force increases as the jaw opens (29–34% increase between the narrowest and widest gapes at the incisors), the absolute force produced by these muscles increases even though their relative contribution reduces. Being able to generate a high incisor bite force at wide gape is likely to be important for a species such as the grey squirrel that includes relatively large and mechanically resistant objects such as hazelnuts and acorns in its diet [68].

The muscles that contributed the most to maximum bite force, as determined by the percentage decrease in bite force when their activations were set to zero, were the superficial masseter and the anterior deep masseter, as predicted by hypotheses 1 and 2. Each of these muscles was separately responsible for a 24–32.4% reduction in bite force (figure 2), albeit with the superficial masseter displaying a very slightly larger contribution (electronic supplementary material, tables S1 and S2). In the grey squirrel, other muscles also play an important role in the generation of bite force, notably the posterior deep masseter and the anterior ZM, each of which was responsible for around a 12.3–14.7% reduction in bite force. These two muscles contribute slightly less in the red squirrel, with ablation of the posterior deep masseter leading to between 8.9% and 11% bite force reduction, and ablation of the anterior ZM reducing bite force by just 4–5%.

Our finding that the superficial masseter is the greatest contributor to both incisor bite force is in line with previous EMG studies on hamsters [34] and guinea pigs [35] that showed this muscle to be one of the most highly activated during jaw closing (along with the deep masseter and medial pterygoid). Given the large size of this muscle, it is perhaps not surprising it plays such a central role in the elevation of the mandible and the power stroke of biting. However, the mechanical advantage of the superficial masseter enables it to be highly efficient, contributing to a much greater proportion of bite force than would be expected from its relative mass (cf. table 1, figures 2 and 3). Previous research has suggested that the moment of the superficial masseter increases with decreasing gape [24], but the results here indicate the opposite, i.e. increasing absolute force as the gape widens. This contradiction is likely a product of the simplified two-dimensional vector representation of muscles in earlier studies compared to the more complex three-dimensional representation of the superficial masseter in our model, which takes into account how it wraps around deeper parts of the masseter.

In sciuromorphous rodents, such as squirrels, and myomorphous rodents, such as rats and mice, the anterior deep masseter has an expanded cranial attachment site that extends on to the rostrum in front of the orbit [17,50,51,69]. Previous work using two-dimensional vector diagrams has suggested that the anterior deep masseter is particularly efficient at producing force at the incisors in both sciuromorphs [10] and myomorphs [24,28], owing to the nearly vertical orientation of its fibres and its rostral position on the skull. This is supported by the results generated by our squirrel MDA models.

The medial pterygoid was revealed to have a relatively low contribution to maximum incisor bite force. For example, ablation of this muscle only resulted in a 6.5–12.4% decrease in incisor bite force in the grey squirrel, and a 6.2–8.2% reduction in the red squirrel (figure 2 and electronic supplementary material, tables S1 and S2). This is despite the medial pterygoid being a relatively large component of the masticatory musculature, accounting for 12.4–18.7% of the total adductor muscle force (table 1). This is likely a result of both the origin and insertion sites being located posteriorly on the cranium and mandible, close to the TMJ, thus conferring a small moment. It should be noted that the performance of the medial pterygoid increases as the jaws open and lengthen the moment arm of this muscle, as shown in figure 2. Given the orientation of the medial pterygoid, an important function of this muscle, alongside the generation of bite force, may be retraction of the mandible. Additionally, the medial pterygoid may have a role in counterbalancing the lateral pull of the masseter, which would otherwise tend to evert the ventral margin of the lower jaw [24] and generate high tensile forces at the symphysis [13,26].

Ablation of the temporalis muscle consistently only resulted in a low reduction of incisor bite force (figure 2). This low contribution to bite force, although in line with our third hypothesis, might at first glance seem quite surprising, as superficially it can appear to be quite a large muscle. However, relatively long muscle fibres lead to this muscle only contributing a small percentage of the total adductor muscle force (7.3–14.2%; table 1), and small moment arms reduce this relative contribution even further, especially at wider gapes. Previous research has hypothesized that the primary role of the temporalis may be to retract the lower jaw [24]. This conclusion was not supported by the analysis of non-maximal molar biting with a virtually ablated temporalis which instead slightly increased posterior movement of the working side condyle. This may be a result of modelling the non-maximal biting at a moderate gape (12–15°), which rotates the coronoid process inferiorly and posteriorly, thus bringing the anterior fibres of the temporalis into a vertical orientation. It will also tend to wrap the more posterior fibres around the zygomatic process of the temporal bone, again generating a more vertical direction of pull. Notwithstanding these results, a role in mandibular retraction for the temporalis is still quite likely when the jaws are near occlusion, given the orientation of the posterior fibres.

Overall, the temporalis was found to reduce condylar movement during premolar biting (although not during bites on the distalmost molar). A similar stabilizing role for the temporalis during molar chewing has also been found in guinea pigs [35] and mice and voles [25]. However, it should be noted that several previous descriptive studies of squirrel jaw adductors have split the temporalis into medial and lateral parts [50,51]; or anterior, posterior and suprazygomatic parts [69,70]. Owing to the difficulty of representing a muscle originating from a fascia, rather than bone, the temporalis in the MDA model here was represented as a single unit, but given the variation in the orientation of the muscle fibres (approximately 90°), this simplification may obscure the different roles of different parts of the temporalis. For instance, Hiiemae [24] and Gorniak [34] both suggested that the anterior temporalis is a powerful elevator of the jaw, while the posterior temporalis is much weaker but important in retraction of the mandible (as noted above).

The lateral pterygoid is particularly unusual among the grey squirrel masticatory muscles, as our model indicates that its ablation barely affects bite force at all, despite comprising 6–9% of total adductor muscle force. This is likely a result of the lateral and near-horizontal orientation of the muscle fibres (figure 1). This suggests that the main function of the lateral pterygoid may not be to assist with vertical jaw closing, but instead this muscle may have a role in generating transverse jaw movements [27], in mandibular protraction [33,34], or even in jaw opening, as has been suggested for the springhare [36].

### 4.1. Limitations

As with any modelling technique, this MDA has limitations which must be acknowledged when interpreting the outputs of the simulation. One limitation is associated with the muscle data used as an input to the grey squirrel model. As noted in the methods, this muscle force data was derived from a formalin-fixed and iodine-stained specimen [18] and must therefore be treated with caution owing to potential shrinkage of the muscles. However, the close match between the predicted incisor bite force and that derived from the regression equation appears to indicate that the total muscle force applied to the model (table 1) was a close approximation. Additionally, the grey squirrel specimen from which muscle data was derived was fixed with its molar teeth almost in occlusion. Thus, our understanding of fibre orientation is strictly limited to that jaw position and does not take into account any stretching or distortion of aponeuroses at wider gapes. However, this is likely to be a minor error given the broad approximation of each muscle as 3–8 strands (table 1).

Another limitation relates to the motion of the jaw in the simulations. To the authors' knowledge, no kinematic data for squirrel jaws during feeding exists in the published literature. Therefore, the movement of the lower jaw in the model presented here was either restricted to simple rotation around the temporo-mandibular joint (as in the maximal incisor biting simulations), or allowed the jaw to follow any desired movement based on the muscle lines of action (used for the molar shearing analyses). Thus, while the results here are useful for understanding the relative contribution of each masticatory muscle to certain jaw movements, *in vivo* kinematic data (e.g. from XROMM [71]) would help to refine the model and more accurately replicate the complete feeding cycle in red and grey squirrels.

Notwithstanding the lack of knowledge of detailed muscle data or complex jaw kinematics, overall, the results here provide the first step towards understanding how each masticatory muscle can contribute to different jaw movements in the grey squirrel. Further analysis, both virtual and experimental, is needed to determine whether the results here can be generalized to other sciurids or even further to sciuromorphous rodents.

## 5. Conclusion

The MDA model constructed here was able to replicate incisor biting in the red and grey squirrel, producing bite force magnitudes very similar to those derived by regression equations. As predicted, the superficial masseter and anterior deep masseter were the largest contributors to bite force, but the temporalis had a surprisingly small role in generating bite force. Instead, we propose that the temporalis has a greater role in stabilization of the mandible against the anterior pull of the masseter. The computational modelling approach of MDA has the potential to further our understanding of red and grey squirrel masticatory biomechanics, but more experimental data would help to refine the model, and analyses of other taxa would reveal how consistent the results are across squirrels and other rodents.

## Data Availability

All image data are available for download from Morphosource (www.morphosource.org). Grey squirrel microCT scans (both osteological and with iodine staining): https://www.morphosource.org/concern/biological_specimens/000S11778. Red squirrel osteological microCT scans: https://www.morphosource.org/concern/biological_specimens/000488412. The data are provided in the electronic supplementary material [72].
